# Incidence of shark‐inflicted bite injuries on Australian snubfin (*Orcaella heinsohni*) and Australian humpback (*Sousa sahulensis*) dolphins in coastal waters off east Queensland, Australia

**DOI:** 10.1002/ece3.10026

**Published:** 2023-05-03

**Authors:** Caitlin R. Nicholls, Katharina J. Peters, Daniele Cagnazzi, Daniella Hanf, Guido J. Parra

**Affiliations:** ^1^ Cetacean Ecology, Behaviour and Evolution Lab, College of Science and Engineering Flinders University Adelaide South Australia Australia; ^2^ School of Earth, Atmospheric and Life Sciences University of Wollongong Wollongong New South Wales Australia; ^3^ School of Earth and Environment University of Canterbury Christchurch New Zealand; ^4^ Marine Ecology Research Centre, Faculty of Science and Engineering Southern Cross University Lismore New South Wales Australia; ^5^ O2 Marine Busselton Western Australia Australia; ^6^ Centre for Sustainable Aquatic Ecosystems Harry Butler Institute, Murdoch University Murdoch Western Australia Australia

**Keywords:** behavior, cetaceans, marine mammals, predation, predation risk

## Abstract

The ecology and evolution of prey populations are influenced by predation and predation risk. Our understanding of predator–prey relationships between sharks and dolphins is incomplete due to the difficulties in observing predatory events directly. Shark‐inflicted wounds are often seen on dolphin bodies, which can provide an indirect measure of predation pressure. We used photographs of Australian humpback and snubfin dolphins from north, central, and south Queensland to assess the incidence of shark‐inflicted bite injuries and to examine interspecific differences in bite injuries and their relationship with group sizes, habitat features, and geographical locations characteristic of where these individuals occurred. The incidence of shark‐inflicted scarring did not differ between species (*χ* ^2^ = 0.133, df = 1, *p* = .715), with 33.3% of snubfin and 24.1% of humpback dolphins showing evidence of shark bites when data were pooled across all three study sites. Generalized additive models indicated that dolphins closer to the coast, with greater photographic coverage, and in north Queensland were more likely to have a shark‐inflicted bite injury. The similar incidence of shark‐inflicted wounds found on snubfin and humpback dolphins suggests both are subject to comparable predation pressure from sharks in the study region. Results highlight the importance that habitat features such as distance to the coast and geographical location could have in predation risk of dolphins from sharks, as well as the importance of considering photographic coverage when assessing the incidence of shark‐inflicted bites on dolphins or other marine animals. This study serves as a baseline for future studies on shark‐dolphin interactions in Queensland and into how predation may influence dolphin habitat usage, group living, and behavior.

## Graphical abstract

This study used photographs of Australian humpback and snubfin dolphins from north, central, and south Queensland to assess the incidence of shark‐inflicted bite injuries on these species and to examine interspecific differences in bite injuries and their relationship with group sizes, habitat features and geographical locations characteristic of where these individuals occurred. The results show that both species are subject to predation from sharks, predation pressure is similar across the two species, and individuals are more likely to have a shark‐inflicted injury if they occur closer to the coast, in Townsville, and have greater photographic coverage. This highlights the importance that habitat features such as distance to the coast and geographical location could play in predation risk of dolphins from sharks, as well as the importance of considering photographic coverage when assessing the incidence of shark‐inflicted bites on dolphins or other marine animals.

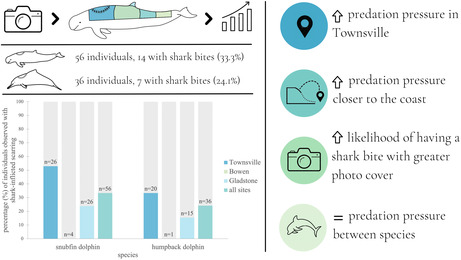

## 1 INTRODUCTION

Predation and predation risk (i.e., the probability of being killed by a predator) can influence the ecology, evolution, behavior, population dynamics, and community structure of prey populations (Heithaus et al., 2017; Heithaus & Dill, 2006; Holt et al., 2008; Kiszka et al., 2011; Wirsing et al., 2014; Wirsing & Ripple, 2011). Aside from the density‐mediated lethal effects predators have on prey through killing or consumption, trait‐mediated effects of predation influence prey behavior and life‐history traits associated with anti‐predatory defenses (Cresswell, 2008; Lima, 1998; Lima & Dill, 1990; Preisser et al., 2005). Thus, both lethal and non‐lethal predation shape community composition (Menge, 1976; Vance, 1979), trophic cascades (Burkholder et al., 2013; Heithaus et al., 2012; Myers et al., 2007; Schmitz et al., 2000), species' coexistence (Parra, 2006; Rosenzweig, 1981) and biodiversity (Glen & Dickman, 2005; Ritchie & Johnson, 2009). Although there have been many studies on lethal and non‐lethal predation effects in the terrestrial environment (Banks et al., 2000; Korpimäki, 1985; Mills & Shenk, 1992; Norrdahl & Korpimäki, 1995), there is a lack of quantitative data and a knowledge gap of marine predator–prey interactions due to the difficulty of observing such relationships in the marine environment.

Marine mammals are apex and mesopredators and thus many species have relatively few natural predators. However, sharks and killer whales (*Orcinus orca*) are natural predators of most marine mammals, with growing evidence that several shark species represent a major threat, particularly to small cetaceans such as dolphins and porpoises (Heithaus, 2001a; Jefferson et al., 1991; Melillo‐Sweeting et al., 2021; Smith et al., 2018). Aside from occasional direct observations of shark attacks (Connor & Heithaus, 2006; Corkeron et al., 1987; Mann & Barnett, 1999), evidence of successful or attempted shark predation on dolphins usually comes from stomach‐contents analysis (Ebert, 2002; Heithaus, 2001b; Lowe et al., 1996; Stein, 1977) and the presence of shark‐inflicted scarring on the bodies of delphinids (Heithaus, 2001b; Melillo‐Sweeting et al., 2021; Smith et al., 2018). The presence of shark‐inflicted wounds and scars on live individuals can provide an indirect measure of predation pressure, representing failed predation attempts, and provide an estimate of the frequency of shark attacks (Heithaus, 2001b; Smith et al., 2018). Studies on the frequency of shark‐scarring among dolphin species of similar size and similar habitats have provided useful insights into shark/dolphin interactions in multiple species of dolphins, including bottlenose (*Tursiops aduncus*; Heithaus, 2001b; Smith et al., 2018; *T. truncatus*; Cockcroft et al., 1989; Melillo‐Sweeting et al., 2021; Wilkinson et al., 2017), Australian snubfin (*Orcaella heinsohni*) and humpback (*Sousa sahulensis*) (Smith et al., 2018), Indian‐Ocean humpback (*Sousa plumbea*) (Cockcroft et al., 1991), and Atlantic spotted dolphins (*Stenella frontalis*) (Melillo‐Sweeting et al., 2014, 2021).

The Australian snubfin dolphin and the Australian humpback dolphin (hereafter, snubfin dolphin and humpback dolphin, respectively) are small (<3 m long) coastal delphinids endemic to northern Australia and Papua New Guinea (Beasley et al., 2005; Hanf et al., 2022; Jefferson & Rosenbaum, 2014; Parra & Cagnazzi, 2015; Stacey & Leatherwood, 1997). In Australia, both species live sympatrically in rivers, estuaries, and coastal waters from Western Australia to Queensland (Beasley et al., 2005; Hanf et al., 2022; Jefferson & Rosenbaum, 2014; Parra & Cagnazzi, 2015; Stacey & Leatherwood, 1997). Both species are currently listed as *Vulnerable* by the Queensland Nature Conservation Act and by the IUCN Red List of threatened species due to their coastal distribution, small population size, low genetic diversity, and the slow life history common in delphinids (Parra, Cagnazzi, & Beasley, 2017; Parra, Cagnazzi, Perrin, & Braulik, 2017). In coastal waters off east Queensland, both species use shallow, coastal‐estuarine waters extensively; however, snubfin dolphins use shallower waters (1–2 m) and seagrass meadows and occur closer to river mouths (Parra, 2006). The species also exhibit differences in grouping patterns, with snubfin dolphins forming larger and more stable groups than humpback dolphins (Parra et al., 2011).

The coastal waters in which these two species reside are also inhabited by several shark species known to prey on small cetaceans. In Queensland, tiger (*Galeocerdo cuvier*), bull (*Carcharhinus leucas*), and white (*Carcharodon carcharius*) sharks overlap in spatial distribution with snubfin and humpback dolphins (Green et al., 2009; Heithaus, 2001b; Heithaus et al., 2017; Monteiro et al., 2006). The probability of predation of small dolphins by sharks is ultimately influenced by the predator's ability to encounter, ambush, and overpower its prey, and the prey's ability to detect, avoid, and escape its predator (Heithaus et al., 2009; Martin & Hammerschlag, 2012). Prey species have evolved a variety of strategies to combat the threat of predation, including active defense (fight or flight; Lima, 1998; Lima & Dill, 1990), grouping (Clark & Mangel, 1986; Norris & Dohl, 1980) and predator avoidance through changes in habitat use (Heithaus & Dill, 2002, 2006). There are, however, environmental factors such as water depth (Heithaus & Dill, 2002; Long & Jones, 1996) and turbidity (Heithaus, 2001b; Turesson & Brönmark, 2007) influencing the preys' detection abilities and the predators' ambush abilities, and thus ultimately affecting the success of predation attempts by sharks (Martin & Hammerschlag, 2012). Similarly, habitat characteristics are also likely to influence predation success. Waters adjacent to the coast could have a higher density of predators and hinder the detection ability of dolphins (Cameron, 2010; Heithaus, 2001a; Heithaus et al., 2002, 2007; Meyer et al., 2009), and hence pose a more dangerous environment for dolphins. Habitats close to estuaries and river mouths may be more dangerous habitats for dolphins due to the spatial overlap with predatory species frequenting this environment such as bull sharks (Heupel & Simpfendorfer, 2008; Melillo‐Sweeting et al., 2021). Additionally, anthropogenic factors such as hunting and culling of sharks might also influence predation of dolphins by sharks, altering the composition of both predator and prey populations and encounter rates (Baum et al., 2003; Holmes et al., 2012).

**FIGURE 1:**
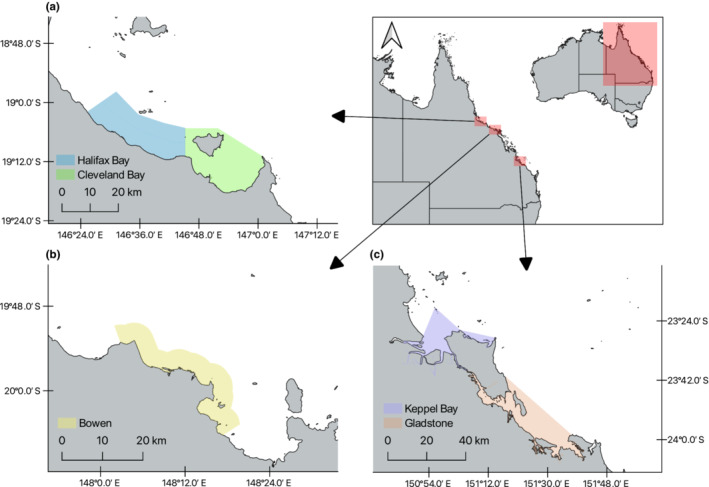
Location of study sites in (a) north, (b) central, and (c) south Queensland, Australia, used to survey Australian snubfin (*Orcaella heinsohni*) and humpback (*Sousa sahulensis*) dolphins.

In this study, we used photographic evidence of shark‐inflicted scarring on individual snubfin and humpback dolphins from northern (Cleveland Bay and Halifax Bay), central (Bowen), and southern (Keppel Bay and Gladstone) Queensland, Australia (Figure 1) to (1) assess the prevalence of shark‐bite scars on snubfin and humpback dolphins; (2) assess if shark bite presence on dolphins differs among dolphin species, study sites and environmental variables (water depth, distance to coast, distance to estuary) associated with dolphin habitat use; and (3) identify which dorsal region where most shark‐inflicted scarring occurs. Based on existing knowledge of predator–prey relationships, relative shark abundance (i.e., shark catches per unit effort), and the ecology of both delphinids and sharks, we predicted that the incidence of shark bites on dolphins would be greater in (1) northern Queensland as this area tends to have a larger shark population (indicated by higher catches per unit effort) than southern areas and thus an expected higher dolphin‐shark encounter rate; (2) shallower water and waters close to the coast and estuaries due to the preference for such environments by predatory shark species, hence higher encounter rate; (3) greater for snubfin dolphins due to their habitat preference for shallower water and waters close to estuaries; (4) greater with more photographic coverage due to the increased likelihood of observing a shark bite; and (5) less incidence of shark bites with increasing group size as this can improve detection of predators or dilute predation risk.

## 2 METHODS

### 2.1 Study sites

To measure the incidence of shark‐inflicted bite injuries on snubfin and humpback dolphins, we used digital photographs of the two species collected across three study sites in Queensland (north, central, and south, Figure 1).

The north study site (~780 km^2^) included Cleveland Bay and Halifax Bay. These sites are shallow, averaging ~10 m, with Cleveland Bay making up the entrance to the Port of Townsville, the third largest seaport in Queensland. The central study site was a 241 km^2^ site off Bowen, including the entrance to the Port of Gladstone, the second largest seaport in Queensland. The south site (1133 km^2^) included Keppel Bay and coastal waters off Gladstone, with both the central and south study sites having maximum depths of ~15 m. We chose these sites because they contain distinct populations of both snubfin and humpback dolphin species based on genetic data and the representative ranges of each species (<500 km^2^; Cagnazzi, 2010; Parra, 2006; Parra et al., 2018).

### 2.2 Data collection

#### 2.2.1 Surveys

Data were collected from ongoing monitoring and research across the study regions between 2014 and 2021 (Table A1) following standard procedures for capture‐recapture studies of inshore dolphin species (Cagnazzi et al., 2011; Parra et al., 2006 for further details). When an individual dolphin or a dolphin group (defined as dolphins within 100 m of any other member and involved in similar behavior; Parra et al., 2011) was sighted, information regarding the species identity, location (latitude and longitude), group size, age composition, behavior, and spatial cohesion were recorded. Individuals were then photographed by two photographers using digital, single‐lens reflex cameras fitted with 50–500 mm telephoto zoom lenses, with photos taken as close and parallel to the animal's dorsal fin and body as possible. Water depth (m) was recorded using the vessel's echosounder at the initial location of the dolphin sighting.

#### 2.2.2 Photo‐identification of dolphins

Photographs of the dorsal side of dolphins identified individuals where possible, primarily using the dorsal fin shape, nicks, and scars (Würsig & Würsig, 1977), as well as loss of pigmentation in the upper region of the dorsal fin (Hunt et al., 2017). Only photographs considered excellent or good quality of dorsal fins with distinctive markings were used for the identification of individuals, development of the catalog and analysis (Parra et al., 2011; Würsig & Jefferson, 1990). Images were then checked using DISCOVERY software (Gailey & Karczmarski, 2012) to be matched with individuals in the catalog. Only marked individuals in the photo‐identification catalogs were used for the analysis of the evidence of shark bites to ensure each individual was only counted once (see Parra et al., 2006 for further details).

#### 2.2.3 Presence of shark bite scars

**FIGURE 2:**
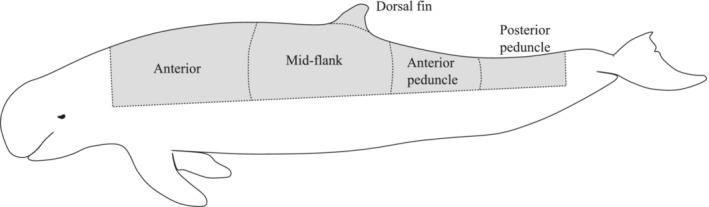
Outline sketch of an Australian snubfin dolphin (*Orcaella heinsohni*) demonstrating the separation of body regions (anterior, mid‐flank, dorsal fin, anterior peduncle, and posterior peduncle; adapted from Smith et al., 2018, as described in Scott et al., 2005) used to determine the locations of shark bites.

We reviewed the capture history of each individual dolphin in the photo‐identification catalogs to source multiple images of each individual's dorsal region and for assessments of shark‐inflicted scarring. Scarring attributed to sharks is generally crescent‐shaped, jagged, and consisted of widely spaced tooth marks (Heithaus, 2001b; Scott et al., 2005; Smith et al., 2018). In the analysis, we did not include scarring that could not be clearly attributed to sharks, such as notches, linear scars and narrowly spaced, shallow rake marks. When shark‐inflicted scars were identified, they were assigned to the body region they covered (Figure 2) and the respective side of the animal (left or right). If individuals had shark bites in more than one region, one region was selected randomly to include in the analysis. We attempted to identify the species of shark responsible for the scarring using the conformation of wounds and spacing between teeth, however, decided against including this due to the unreliability of such methods.

**FIGURE A1:**
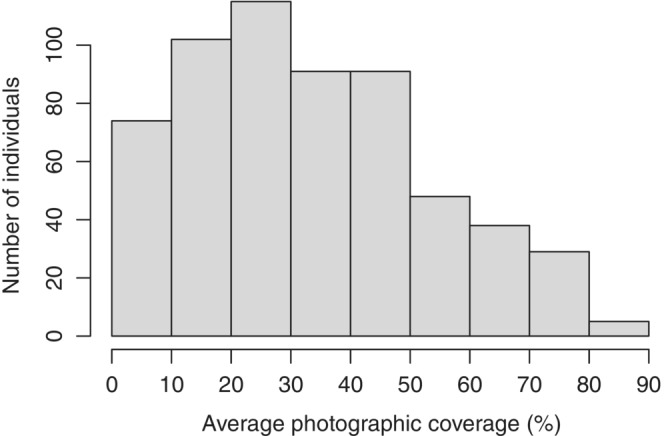
Histogram of average photographic coverage of individual Australian snubfin (*Orcaella heinsohni*) and Australian humpback dolphins (*Sousa sahulensis*).

We estimated the photographic coverage of the dorsal side of each individual in the photo‐identification catalogs, regardless of the presence of shark bites, by recording which body regions (as indicated in Figure 2) had been photographed, and then calculating a percentage of the dorsal side of each individual photographed. Photographic coverage was explored as a variable (Figure A1), and then only individuals with ≥60% of their dorsal body photographed were selected for analysis to standardize the comparison of shark‐inflicted wounds among individual dolphins and to minimize bias in the incidence of shark bites towards individuals with greater photographic coverage.

### 2.3 Data analysis

All analyses were done in R version 4.0.2 (R Core Team, 2022).

#### 2.3.1 Univariate analysis

Average water depth, group size, distance to coast, distance to estuary, and photographic coverage were calculated using the average of all sightings of that individual across the study period (using the capture history) to ensure that a representative range of habitat use of each individual was reflected (Table 1). We examined the relationship between each explanatory variable (Table 1) and the incidence of shark‐inflicted wounds using a chi‐squared with Yates' continuity correction test to assess differences in shark bite incidence between dolphin species and study sites. A Fisher's exact test for count data was used to compare differences in shark bite incidence across the left and right side, as well as the different body regions of dolphins. Randomization tests were used to compare the mean of each predictor variable (average water depth, group size, distance to coast, distance to estuary, and photographic coverage) between individuals with and without shark‐inflicted scarring.

**TABLE 1:** Description of predictor variables, including the abbreviations used and a description of how they were calculated, used in modeling of shark bite prevalence on Australian snubfin (*Orcaella heinsohni*) and Australian humpback dolphins (*Sousa sahulensis*).

Predictor variable	Description
Dolphin species	Species of dolphin was determined by looking at the morphology of each individual and determined to be either Australian snubfin or humpback dolphins
Group size	Group size was recorded by estimating the number of individuals in each group (defined as dolphins within 100 m of any other member and involved in similar behavioral activities; Hunt et al., 2017)
Photographic coverage	Average photographic coverage of individuals was calculated by recording which body regions had been photographed, and then calculating a percentage of each individual photographed
Water depth	Average water depth was calculated using the average of all sightings of that individual across the study period (using the capture history) to ensure that a representative range of habitat use of each individual was reflected
Distance to coast	Distance to coast was calculated as Euclidean distance using the coordinates for the sightings and the cost distance function in ArcGIS Pro version 2.8.0 (ESRI, 2022). Average distance to coast was then calculated using the average of all sightings of that individual across the study period (using the capture history) to ensure that a representative range of habitat use of each individual was reflected
Distance to estuary	Distance to estuarine waters was calculated the same as distance to coast
Study site	Study site refers to the site from which the data is collected, including the north, central, and south site

#### 2.3.2 Generalized additive modeling

We used generalized additive modeling (GAM; Hastie & Tibshirani, 2017) to model the relationships between the presence of shark‐inflicted scarring and a suite of predictor variables including dolphin species, group size, photographic coverage, water depth, distance to coast, distance to estuary, and study site (Table 1). As the central study site only had five individuals with ≥60% photographic coverage, this site was excluded from models to avoid model overfitting. Correlation between variables was checked using Spearman's rank correlation test and by calculating the variance inflation factor using the udsm package (Naimi, 2015), with no correlation found between the variables. We standardized numerical data prior to analysis using the STANDARDIZE function in Excel (Microsoft Corporation, 2022), returning a normalized value (*z*‐score), to allow for interpretation of the relative strength of parameter estimates in the averaged model (Grueber et al., 2011). A total of 128 GAM models were built with binomial distribution and a logit link function using the mgcv package (Wood, 2001), including the null model, using all possible combinations of predictor variables. To prevent overfitting, gamma was set to 1.4 (Wood, 2017). Models were ranked using Akaike's information criterion corrected for small sample size (AIC*c*) and final models were checked for patterns in the residuals. We adopted an information‐theoretic approach (described by Burnham & Anderson, 2002) and averaged the top competing models (ΔAIC*c* < 1, as recommended by Burnham & Anderson, 2002). The sum of Akaike weights was then calculated for averaged top models using the qpcR package (Spiess, 2018) to determine the importance of the predictor variables.

## 3 RESULTS

Boat‐based surveys across the three study sites resulted in a total of 1531 observations of dolphins and 593 photo‐identified individual dolphins (248 snubfin dolphins and 345 humpback dolphins), of which 72 (37 snubfin and 35 humpback dolphins) had shark‐inflicted wounds on their dorsal area (Table A1). Of these 593 individual dolphins, 92 (56 snubfin and 36 humpback dolphins) had photographic coverage of ≥60% of their dorsal body, with 21 of these (14 snubfin and 7 humpback dolphins) showing shark‐inflicted scarring (Table A1). Most animals included in the analysis were sighted several times (mean ± SE = 3.2 ± 0.24, range = 1–10 sightings), and throughout the study period (mean ± SE = 224.7 ± 28.4, range = 0–765 days; Table A2).

### 3.1 Incidence of shark‐inflicted scars

**FIGURE 3:**
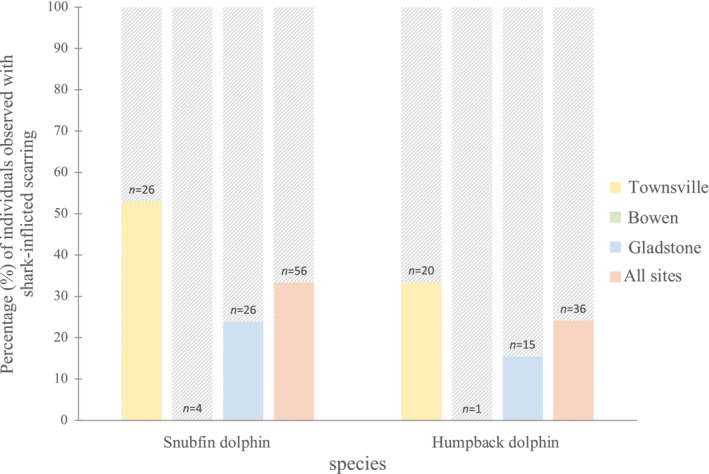
Prevalence of shark bites on Australian snubfin (*Orcaella heinsohni*) and Australian humpback dolphins (*Sousa sahulensis*) with photographic coverage of ≥60% from north (Halifax Bay and Cleveland Bay), central (Bowen), and south (Keppel Bay and Gladstone) Queensland, as well as all sites combined. *N* represents sample size from each study site.

The incidence of shark‐inflicted scarring on individuals with photographic coverage of ≥60% did not differ between species for combined data (shark‐wound prevalence for snubfin dolphins = 33.3%, *n* = 14, humpback dolphins = 24.1%, *n* = 7; *χ* ^2^ = 0.133, df = 1, *p* = .715, Figure 3). Similarly, there was no evidence of a difference in the incidence of shark‐inflicted scarring between species within each study site (north: *p* = .7044, south: *p* = .958, Figure 3).

**FIGURE A2:**
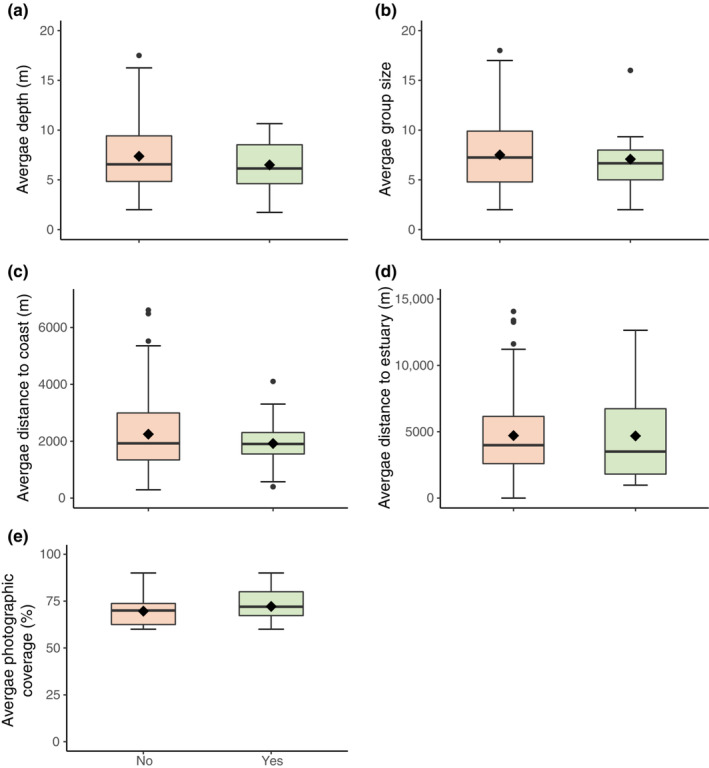
Average (a) water depth, (b) group size, (c) distance to coast, (d) distance to estuary, and (e) photographic coverage of individual Australian snubfin (*Orcaella heinsohni*) and Australian humpback dolphins (*Sousa sahulensis*) with photographic coverage of ≥60% without shark‐inflicted scarring photographed (no) and with shark‐inflicted scarring photographed (yes) from north (Halifax Bay and Cleveland Bay), central (Bowen), and south (Keppel Bay and Gladstone) Queensland. Whiskers extend 1.5× the interquartile range from the hinge, the boxes represent the central 75% interquartile range, horizontal lines represent medians, and diamonds represent means.

The average water depth, group size, distance to coast, and distance to estuary at which individual dolphins with photographic coverage of ≥60% with and without shark scars were sighted, as well as their photographic coverage, did not differ (randomization test, all *p* > .05; Table 2, Figure A2).

**TABLE 2:** Mean, standard deviation (SD) and randomization test statistics of predictor variables associated with Australian snubfin (*Orcaella heinsohni*) and Australian humpback dolphins (*Sousa sahulensis*) with photographic coverage of ≥60% with and without shark‐inflicted scarring in north (Halifax Bay and Cleveland Bay), central (Bowen), and south (Keppel Bay and Gladstone) Queensland study sites.

Variable	Shark bite scar	Mean	SD	*p* Value
Depth (m)	Yes	6.50	2.56	.166
	No	7.37	3.46	
Group size	Yes	7.08	3.57	.319
	No	7.50	3.82	
Distance to coast (m)	Yes	1919.78	986.30	.167
	No	2246.93	1463.38	
Distance to estuary (m)	Yes	4681.34	3450.63	.462
	No	4707.40	3264.99	
Photo coverage (%)	Yes	72.15	8.54	.102
	No	69.63	7.79	

### 3.2 Generalized additive modeling

GAM modeling of individuals with ≥60% photographic coverage in the north and south study site returned eight models within 1 delta AIC*c*, including the null model. The top models (ΔAIC*c* < 1.0) are listed in Table 3.

**TABLE 3:** Model formula, % DE, ΔAIC*c* and AIC*c* weights (wAIC*c*) of the eight top models (ΔAIC*c* < 1.0) of shark bite prevalence on Australian snubfin (*Orcaella heinsohni*) and Australian humpback dolphins (*Sousa sahulensis*) using only individuals with ≥60% photographic coverage.

Model	Formula	% DE	ΔAIC*c*	*w*AIC*c*
GAM127	shark_bite ~ site	0.022	0.000	0.040
GAM0	shark_bite ~ 1	0.000	0.056	0.039
GAM116	shark_bite ~ s(av_dist_coast, *k* = 3) + s(av_photo_cover, *k* = 3)	0.063	0.248	0.035
GAM126	shark_bite ~ s(av_photo_cover, *k* = 3)	0.018	0.439	0.032
GAM117	shark_bite ~ s(av_dist_coast, *k* = 3) + site	0.058	0.550	0.030
GAM98	shark_bite ~ s(av_dist_coast, *k* = 3) + s(av_photo_cover, *k* = 3) + site	0.083	0.559	0.030
GAM120	shark_bite ~ s(av_photo_cover, *k* = 3) + site	0.037	0.716	0.028
GAM124	shark_bite ~ s(av_dist_coast, *k* = 3)	0.034	0.723	0.028

**FIGURE 4:**
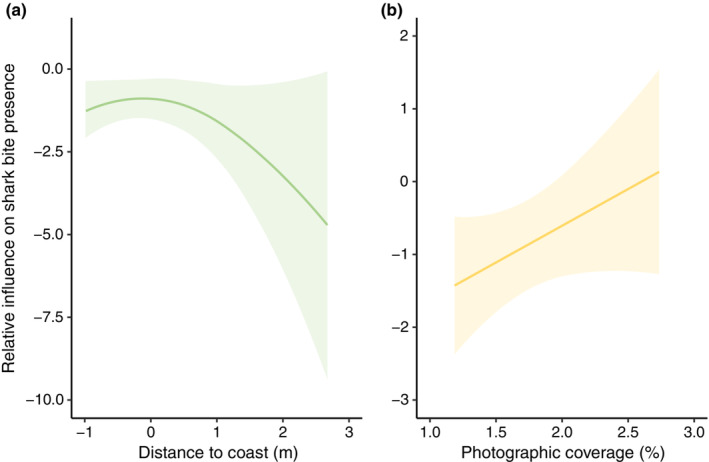
Partial effect plots generated for each variable shown to be influential in the eight top (delta AIC*c* < 1) models, relating the relative influence of (a) distance to coast (m) (negative), and (b) photographic coverage (%) (positive) on sharkbite presence. Solid lines are the fitted linear models. Shaded areas are approximate 95% confidence intervals. Data were standardized, representing the number of standard deviations a given data point is from the mean.

In general, there was an increase in the likelihood of an individual having a shark bite with increased photographic coverage and decreased distance to coast (Figure 4), with individuals being more likely to have shark‐bite injuries in the north study site. The sum of weights of the averaged top models (ΔAIC*c* < 1.0) suggested that for individuals with ≥60% photo cover, the presence of a shark‐inflicted bite was best predicted by study site, average photographic coverage, and average distance to coast (Table 4). The deviance explained was extremely low for all models, suggesting that the variables included here are not sufficient to explain our data and there are other factors at play.

**TABLE 4:** Sum of weights of predictor variables from averaged models of shark bite prevalence on Australian snubfin (*Orcaella heinsohni*) and Australian humpback dolphins (*Sousa sahulensis*) including only individuals with photographic coverage of ≥60%.

Predictor variable	Sum of weights
Site	0.489
s(av_photo_cover, *k* = 3)	0.478
s(av_dist_coast, *k* = 3)	0.471

### 3.3 Location of shark bites

**FIGURE 5:**
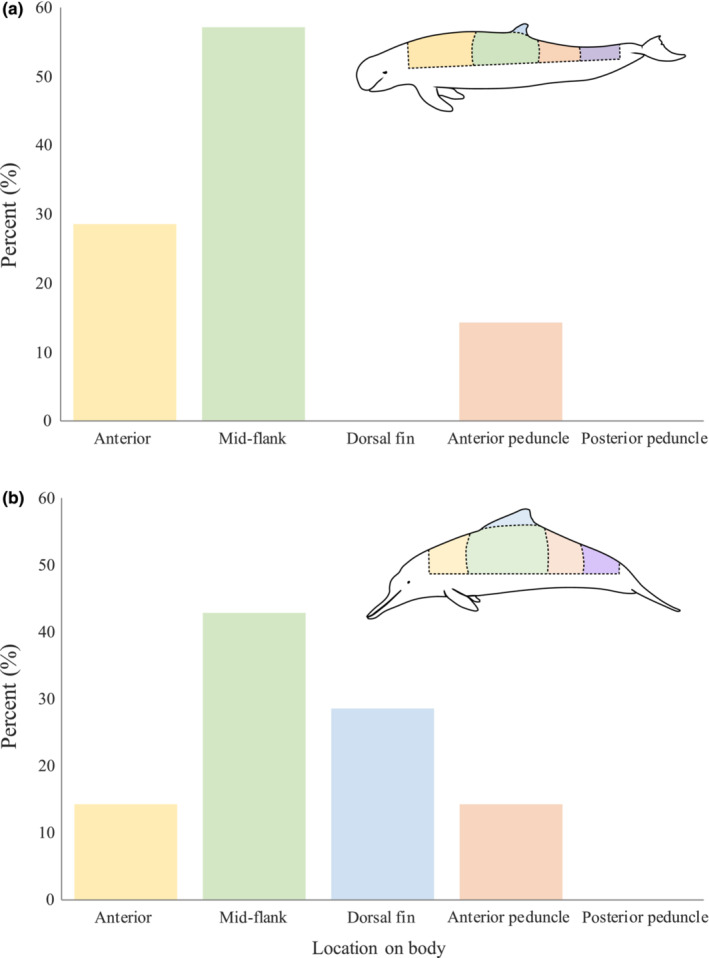
Percentage (%) of shark‐inflicted scarring on each body region (anterior, mid‐flank, dorsal fin, posterior peduncle, and anterior peduncle) of (a) Australian snubfin (*Orcaella heinsohni*) and (b) Australian humpback dolphins (*Sousa sahulensis*) with ≥60% photographic coverage from Cleveland Bay, Halifax Bay, Bowen, Keppel Bay and Gladstone, Queensland.

There were no differences in the incidence of shark‐inflicted bite presence between the left and right side of individuals (snubfin: *p* = .07, humpback: *p* = 1); therefore, we merged data for both sides and only focused on dorsal body regions. The distribution of shark‐inflicted bite injuries on snubfin dolphins with photographic coverage of ≥60% was not random (*p <* .001), with most shark‐inflicted scarring in the mid‐flank region (57.2%), followed by the anterior (28.6%) and anterior peduncle (14.3%) region, with no shark wounds photographed in the dorsal fin or posterior peduncle region (Figure 5a). Shark‐inflicted scarring across different body regions of humpback dolphins with ≥60% photographic coverage was random, with shark‐inflicted scarring most prevalent in the mid‐flank region (42.9%), followed by the dorsal (28.6%), anterior peduncle (14.3%) and anterior (14.3%) region, and no shark wounds recorded in the posterior peduncle region (*p* > .05; Figure 5b).

## 4 DISCUSSION

The ecology, evolution, behavior, population dynamics, and community structure of prey populations are influenced largely by predation and predation risk (Heithaus et al., 2017; Heithaus & Dill, 2006; Holt et al., 2008; Kiszka et al., 2011; Wirsing et al., 2014; Wirsing & Ripple, 2011). However, little is known about the behavioral response of dolphins to non‐lethal shark encounters, including how this influences their decisions regarding habitat use, group living, and foraging. Our research is the first assessment of inferred predation risk in relation to environmental variables and grouping patterns of snubfin and humpback dolphins in eastern Australia. The presence of shark‐inflicted scarring to infer predation pressure on dolphins indicates only failed predation attempts (Heithaus, 2001b; Smith et al., 2018), and hence, our observations here should be considered a minimum estimate of predation pressure.

We acknowledge that the small sample sizes in this study may limit the generalisability of the findings and the statistical power of our analysis. Although the sample sizes were small, snubfin, and humpback dolphins occur at very low densities (Parra & Cagnazzi, 2015; Parra, Cagnazzi, & Beasley, 2017; Parra, Cagnazzi, Perrin, & Braulik, 2017), and thus, we believe that the findings provided here represent robust patterns and insights into shark bite prevalence on these species along the east coast of Queensland.

Analysis of shark‐bite scars on the dorsal body of snubfin and humpback dolphins suggests that both species are subject to predation from sharks, that predation pressure is similar across the two species and appears to be influenced by distance to coast and the geographic location along the coast. Additionally, our analysis highlights the importance of considering photographic coverage when assessing the incidence of shark‐inflicted bites on dolphins or other marine animals.

### 4.1 Interspecific differences

Analysis of photographs from the dorsal regions of the body of snubfin and humpback dolphins indicate that both are subject to predation attacks by sharks. We found predation pressure (as inferred from the prevalence of shark‐inflicted bite injuries) to be consistent between the two species in coastal waters of east Queensland, Australia. Interspecific variation in the incidence of shark bites on dolphins could be linked to their habitat use patterns as well as differences in shark abundance, shark sizes, or food availability among study sites. In Queensland, snubfin dolphins prefer shallower waters (1–2 m), occur closer to river mouths, and form larger groups than humpback dolphins (Parra, 2006). Despite slight differences in their habitat preferences, both species' patterns of space use can overlap considerably (i.e., >50%; Parra, 2006). Thus, both species could be facing similar predation risks from sharks resulting in the overall lack of interspecific differences in the incidence of shark‐inflicted scars we observed in this study.

In contrast with our results, Smith et al. (2018) found a higher prevalence of shark bites on snubfin and humpback dolphins in northwestern Australia (snubfin = 72%, humpback = 46%) than what we found in Queensland (snubfin = 33%, humpback = 24%), despite the similar methodology used in both studies. In Queensland, large sharks have been heavily culled as part of the Queensland Shark Control Program since 1962 (Paterson, 1990), and there has been a decline in the number and average size of sharks because of culling (Holmes et al., 2012). In northwestern Australia no major shark culling programs are in place; a vast area (~0.8 million km^2^) has been closed to commercial shark fishing since 1993 and 2005, and tiger sharks were the most commonly caught species (58% of all individuals caught) in scientific longline surveys between 2002 and 2017 (Braccini et al., 2020). Therefore, snubfin and humpback dolphins in northwestern Australia may be subject to higher shark predation risks than those in Queensland and, thus, the difference in shark bite prevalence between studies. Furthermore, differences in sample size between the two studies (Western Australia = 152 snubfin and 26 humpback dolphins, Queensland = 56 snubfin and 36 humpback dolphins) may have also contributed to the contrasting findings.

Future research on the habitat use and spatial preferences of snubfin and humpback dolphins in north Western Australia, as well as the shark abundance across different study sites, should elucidate why predation risk of the two species differs between the populations in eastern and northwestern Australia.

### 4.2 Distance to coast

Although distance to coast for individuals with and without shark‐inflicted scarring was not different between the two groups, the likelihood of an individual bearing shark‐inflicted scarring increased the closer it was observed to the coast, supporting the hypothesis that the incidence of shark bites would be greater close to the coast. Coastal regions are productive areas, with combinations of estuary output, nutrient run‐off, and upwelling increasing productivity and food availability in these areas (Webb, 2021). For example, Cleveland and Halifax Bays in the north site are productive mangrove habitats, supporting large populations of teleosts and attracting both sharks and dolphins to feed (Robertson & Duke, 1987, 1990; Simpfendorfer & Milward, 1993). Additionally, due to the abundance of food, sharks often use coastal areas, such as Cleveland Bay, as a nursery habitat (Simpfendorfer & Milward, 1993). More sharks and dolphins in areas close to the coast would increase encounter rate (Heithaus et al., 2009) and presumably the risk of predation on dolphins. Therefore, it could be expected that dolphins occurring closer to the coast would be more likely to have shark‐inflicted scarring as they are exposed to greater predation pressure in these areas. It is also possible that the selection of areas close to the coast happens after predation attempts have occurred; however, these species generally use shallow, estuarine, coastal areas along the east coast of Queensland (Parra, 2006).

### 4.3 Study site

Snubfin and humpback dolphins were more likely to have shark‐inflicted scarring in the northern study site. This may be due to differences in the relative shark abundance in these areas, with the abundance of predatory species such as tiger sharks having declined more in south Queensland compared with north and central Queensland (Holmes et al., 2012). Additionally, the north site (Cleveland Bay and Halifax Bay) is recognized as a nursery area for predatory species of sharks including tiger sharks (Simpfendorfer, 1992; Simpfendorfer & Milward, 1993). Areas with a higher abundance of predators would pose a greater risk of predation for dolphins due to a higher encounter rate (Heithaus, 2001a), hence it could be expected that dolphins occurring in the north study site would face greater predation pressure and, therefore, have a higher incidence of shark‐inflicted bite injuries. To infer why study site was an influential variable on the likelihood of an individual having a shark‐inflicted bite injury, future studies should assess additional variables for each study site including shark size and abundance, as well as the health of ecosystems and the influence of urbanization and overfishing at each site on shark abundance.

### 4.4 Location of shark bites

**FIGURE A3:**
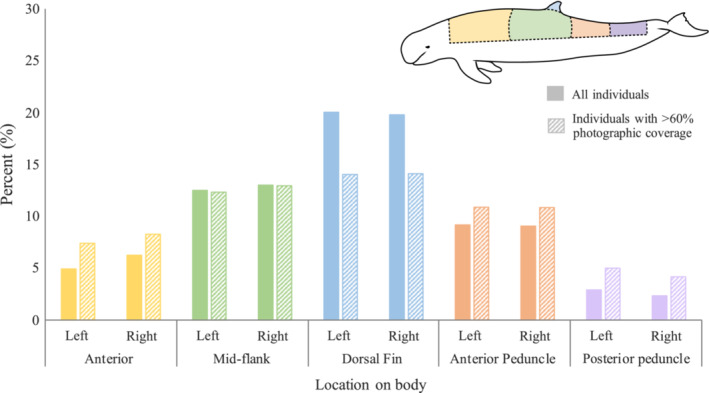
Percentage (%) of each body region (anterior, mid‐flank, dorsal fin, posterior peduncle, and anterior peduncle) photographed of all individuals (dark) and individuals with ≥60% of their dorsal body photographed (striped) for both Australian snubfin (*Orcaella heinsohni*) and Australian humpback dolphins (*Sousa sahulensis*) from Cleveland Bay, Halifax Bay, Bowen, Keppel Bay, and Gladstone.

We found that the majority of shark bites on snubfin and humpback dolphins were in the mid‐flank region, followed by the anterior and anterior peduncle regions. The mid‐flank and dorsal fin regions of dolphins are the most commonly photographed body part due to the surfacing pattern of dolphins, with the remaining dorsal region (e.g., anterior, anterior peduncle, and posterior peduncle) photographed less often (refer to Figure A3 for photographic coverage of individuals). It is possible that fewer bites were observed in the anterior and posterior regions due to lack of photographic coverage of these areas. Furthermore, bites to the anterior and posterior peduncle are more likely to be lethal as they target vital organs and sever the tailstock, immobilizing dolphins and allowing sharks to finalize the kill (Cockcroft et al., 1989; Mann & Barnett, 1999; Smith et al., 2018; Turnbull & Dion, 2012). Therefore, scarring in these areas would not be observed as often on live animals compared with bites on the mid‐flank region, with dolphins more able to escape and recover from bites to this area.

### 4.5 Photographic coverage

Photographic coverage did not differ between individuals with and without shark‐inflicted bite injuries; however, it was included in four of the eight top models, with a positive relationship to the likelihood of an individual having a shark‐inflicted bite injury detected. It would be expected that photographic coverage would influence shark‐bite presence due to the increased likelihood of observing shark‐inflicted scarring if more body regions of the dolphin are observed. Previous studies of shark‐dolphin interactions were able to standardize their data to individuals that had the entirety of their dorsal side photographed (Melillo‐Sweeting et al., 2021; Smith et al., 2018), whereas we were only able to standardize to individuals ≥60% of their body photographed.

Despite only including individuals with photographic coverage ≥60% to minimize the bias of photographic coverage on the likelihood of observing a shark‐inflicted bite injury, the variable was still retained in the top‐ranked models. This indicates that even with standardization, photographic coverage is an important variable that should be taken into consideration when assessing the incidence of shark‐inflicted bites on dolphins and possibly other marine animals.

### 4.6 Additional factors

We found no difference in distance to estuary, water depth, or group size between individuals with and without shark bites. This suggests that dolphins face equal predation pressure across different distances to estuaries, depths, and group sizes in these areas, that dolphins did not change their habitat or grouping behavior after being attacked or that factors other than predation risk are influencing habitat selection and behavior of dolphins, such as prey availability. Additionally, the small sample size of this study may potentially limit the power to detect the influence of these variables on predation pressure.

## 5 CONCLUSION

Predator–prey relationships are complex and influenced by a variety of intrinsic and extrinsic factors (Heithaus, 2001a; Martin & Hammerschlag, 2012). The low deviation explained by the competing GAMs on the incidence of shark bites on snubfin and humpback dolphins indicates that there are additional factors (e.g., size, age, and behavior of both predator and prey species, water turbidity, predator abundance) to those considered in this study that might influence shark‐bite incidence (Heithaus, 2001a; Smith et al., 2018). Despite these limitations, the results of this study offer insights into the predation pressure that both these species are potentially subject to, and species, habitat, and location features that influence shark‐dolphin interactions. This study is the first to assess the occurrence of shark‐bite scarring on snubfin and humpback dolphins in coastal waters off east Queensland, Australia, across different group sizes, habitat features, and locations. Predation and predation risk have a large influence on the ecology and evolution of both predator and prey species; therefore, to decipher how communities are structured and function, we need to understand how predators and prey interact. This study serves as a baseline for shark‐dolphin interactions in Queensland, with further studies of both dolphin and shark populations in these areas needed to provide additional insights into how predation pressure is influencing the behavior, ecology, evolution, population dynamics, and community structure of dolphin populations.

## AUTHOR CONTRIBUTIONS

**Caitlin R. Nicholls:** Conceptualization (supporting); data curation (equal); formal analysis (equal); investigation (equal); methodology (equal); project administration (supporting); software (equal); validation (equal); visualization (lead); writing – original draft (lead); writing – review and editing (lead). **Katharina J. Peters:** Formal analysis (equal); investigation (supporting); methodology (equal); software (equal); supervision (supporting); validation (equal); visualization (supporting); writing – original draft (supporting); writing – review and editing (supporting). **Daniele Cagnazzi:** Data curation (equal); funding acquisition (supporting); project administration (supporting); supervision (supporting); writing – review and editing (supporting). **Daniella Hanf:** Data curation (equal); project administration (supporting); supervision (supporting); writing – review and editing (supporting). **Guido J. Parra:** Conceptualization (lead); data curation (equal); formal analysis (equal); funding acquisition (lead); investigation (supporting); methodology (equal); project administration (lead); resources (lead); software (equal); supervision (lead); validation (equal); visualization (supporting); writing – original draft (supporting); writing – review and editing (supporting).

## CONFLICT OF INTEREST STATEMENT

The authors declare no conflicts of interest.

## Data Availability

Data supporting the results of this study will be archived in https://figshare.com/s/7cd05c413684a19b9e5d.

## APPENDIX 1

**TABLE A1:** Summary of survey years and the number of Australian snubfin (*Orcaella heinsohni*) and Australian humpback dolphins (*Sousa sahulensis*) identified, including the number of those identified with shark‐inflicted bite scarring, for Queensland study sites.

Study area	Sampling site	Survey year (months)	Number of identified individuals^a^				Number of identified individuals with shark bites^a^			
			Snubfin		Humpback		Snubfin		Humpback	
			All^b^	≥60%^c^	All	≥60%	All	≥60%	All	≥60%
North Queensland	Halifax Bay	2019 (June–July)	39	5	48	7	9	2	5	3
		2020 (June–July)	26	12	35	10	7	3	2	2
		2021 (June–July)	20	4	32	5	5	0	1	1
	Cleveland Bay	2019 (June–July)	35	11	19	7	6	4	1	1
		2020 (June–July)	27	13	24	13	7	4	5	5
		2021 (June–July)	17	4	26	11	4	1	4	3
Central Queensland	Bowen	2016 (August)	15	1	37	1	1	0	0	0
		2017 (July)	30	3	24	0	2	0	0	0
South Queensland	Keppel Bay	2014 (May–September)	51	6	54	4	6	1	8	1
		2015 (May–September)	92	23	38	7	9	4	6	2
	Gladstone	2014 (May–August)	0	0	89	5	0	0	14	0
		2015 (May–September)	0	0	100	7	0	0	19	0

^a^ Some individuals were sighted across multiple locations and/or multiple years.

^b^ All individuals regardless of photographic coverage.

^c^ Individuals with photographic coverage of ≥60%.

**TABLE A2:** Individual ID, number of sightings, and the time lag between the first and last sighting of individual Australian snubfin (oh) (*Orcaella heinsohni*) and Australian humpback dolphins (ss) (*Sousa sahulensis*) with photographic coverage ≥60%.

Individual ID	Number of sightings	Time lag between first and last sighting (days)
oh_001_n	3	377
oh_002_n	4	731
oh_004_n	5	734
oh_010_n	8	734
oh_030_n	4	23
oh_032_n	5	396
oh_033_n	3	15
oh_034_n	3	393
oh_036_n	1	0
oh_046_n	4	742
oh_048_n	2	382
oh_051_n	5	713
oh_064_n	1	0
oh_069_n	4	380
oh_073_n	2	25
oh_074_n	3	14
oh_075_n	1	0
oh_078_n	1	0
oh_079_n	3	364
oh_081_n	1	0
oh_082_n	1	0
oh_083_n	2	8
oh_087_n	2	3
oh_089_n	3	335
oh_112_s	2	71
oh_122_s	2	53
oh_133_s	6	407
oh_135_s	1	0
oh_14_s	2	62
oh_15_s	2	16
oh_191_s	2	18
oh_192_s	2	68
oh_208_s	1	0
oh_214_s	2	21
oh_221_s	2	16
oh_222_s	2	21
oh_22212_c	1	0
oh_22321_c	1	0
oh_22323_c	2	1
oh_22327_c	1	0
oh_264_s	1	0
oh_266_s	1	0
oh_269_s	1	0
oh_278_s	1	0
oh_292_s	1	0
oh_295_s	1	0
oh_35_s	2	21
oh_61_s	1	0
oh_69_s	1	0
oh_72_s	4	396
oh_77_s	1	0
oh_81_s	1	0
oh_82_s	4	400
oh_Unk.3_n	1	0
oh_Unk.6_n	2	8
oh_Unk.7_n	2	8
ss_001_n	10	764
ss_002_n	5	402
ss_003_n	8	765
ss_004_n	8	728
ss_006_n	8	763
ss_012_n	6	406
ss_013_n	4	760
ss_029_n	8	731
ss_034_n	2	20
ss_036_n	8	757
ss_042_n	3	396
ss_056_n	8	734
ss_060_n	7	734
ss_074_n	1	0
ss_078_n	1	0
ss_085_n	1	0
ss_089_n	2	364
ss_092_n	1	0
ss_095_n	3	320
ss_107_n	1	0
ss_1361_s	4	434
ss_162_s	4	100
ss_164_s	5	435
ss_169_s	5	100
ss_170_s	8	433
ss_1811_s	5	394
ss_21564_c	1	0
ss_250_s	8	455
ss_274_s	5	430
ss_292_s	4	87
ss_318_s	7	428
ss_389_s	3	100
ss_394_s	2	89
ss_405_s	2	95
ss_439_s	2	56
ss_463_s	5	434

## References

[ece310026-bib-0001] Banks, P. B. , Newsome, A. E. , & Dickman, C. R. (2000). Predation by red foxes limits recruitment in populations of eastern grey kangaroos. Austral Ecology, 25(3), 283–291. 10.1046/j.1442-9993.2000.01039.x

[ece310026-bib-0002] Baum, J. K. , Myers, R. A. , Kehler, D. G. , Worm, B. , Harley, S. J. , & Doherty, P. A. (2003). Collapse and conservation of shark populations in the Northwest Atlantic. Science, 299(5605), 389–392. 10.1126/science.1079777 12532016

[ece310026-bib-0003] Beasley, I. , Robertson, K. M. , & Arnold, P. (2005). Description of a new dolphin, the Australian snubfin dolphin *Orcaella heinsohni* Sp. N. (Cetacea, Delphinidae). Marine Mammal Science, 21(3), 365–400. 10.1111/j.1748-7692.2005.tb01239.x

[ece310026-bib-0004] Braccini, M. , Molony, B. , & Blay, N. (2020). Patterns in abundance and size of sharks in northwestern Australia: Cause for optimism. ICES Journal of Marine Science, 77(1), 72–82. 10.1093/icesjms/fsz187

[ece310026-bib-0005] Burkholder, D. , Heithaus, M. , Fourqurean, J. , Wirsing, A. , & Dill, L. (2013). Patterns of top‐down control in a seagrass ecosystem: Could a roving apex predator induce a behaviour‐mediated trophic cascade? The Journal of Animal Ecology, 82(6), 1192–1202. 10.1111/1365-2656.12097 23730871

[ece310026-bib-0006] Burnham, K. P. , & Anderson, D. R. (2002). Model selection and multimodel inference: A practical information‐theoretic approach (2nd ed.). Springer.

[ece310026-bib-0007] Cagnazzi, D. (2010). Conservation status of Australian snubfin dolphin, Orcaella heinsohni, and Indo‐Pacific humpback dolphin, Sousa chinensis, in the Capricorn coast, Central Queensland, Australia .

[ece310026-bib-0008] Cagnazzi, D. , Harrison, P. L. , Ross, G. J. B. , & Lynch, P. (2011). Abundance and site fidelity of Indo‐Pacific humpback dolphins in the Great Sandy Strait, Queensland, Australia. Marine Mammal Science, 27(2), 255–281. 10.1111/j.1748-7692.2009.00296.x

[ece310026-bib-0009] Cameron, K. E. (2010). Regional variation in tiger shark (Galeocerdo cuvier) abundance and habitat use . 10.25148/ETD.FI10081208

[ece310026-bib-0010] Clark, C. W. , & Mangel, M. (1986). The evolutionary advantages of group foraging. Theoretical Population Biology, 30(1), 45–75. 10.1016/0040-5809(86)90024-9

[ece310026-bib-0011] Cockcroft, V. G. , Cliff, G. , & Ross, G. J. B. (1989). Shark predation on Indian Ocean bottlenose dolphins *Tursiops truncatus* off Natal, South Africa. South African Journal of Zoology, 24(4), 305–310. 10.1080/02541858.1989.11448168

[ece310026-bib-0096] Cockcroft, V. G. , Donovan, G. P. , & Leatherwood, S. (1991). Incidence of shark bites on Indian Ocean humpback dolphins (*Sousa plumbea*) off Natal, South Africa. Cetaceans and Cetacean Research in the Indian Ocean Sanctuary, 3, 277–282.

[ece310026-bib-0012] Connor, R. , & Heithaus, M. (2006). Approach by great white shark elicits flight response in bottlenose dolphins. Marine Mammal Science, 12, 602–606. 10.1111/J.1748-7692.1996.TB00074.X

[ece310026-bib-0013] Corkeron, P. , Morris, R. , & Bryden, M. (1987). Interactions between bottlenose dolphins and sharks in Moreton Bay, Queensland. Aquatic Mammals, 13(3), 109–113.

[ece310026-bib-0014] Cresswell, W. (2008). Non‐lethal effects of predation in birds. Ibis, 150(1), 3–17. 10.1111/j.1474-919X.2007.00793.x

[ece310026-bib-0015] Ebert, D. (2002). Ontogenetic changes in the diet of the sevengill shark (*Notorynchus cepedianus*). Marine and Freshwater Research, 53, 517–523. 10.1071/MF01143

[ece310026-bib-0101] ESRI . (2022). GIS Mapping Software, Location Intelligence & Spatial Analytics. https://www.esri.com/en‐us/home

[ece310026-bib-0016] Gailey, G. , & Karczmarski, L. (2012). DISCOVERY: Photo‐identification data‐management system for individually recognizable animals . http://www.cetaecoresearch.com/research‐software‐discovery.html

[ece310026-bib-0017] Glen, A. S. , & Dickman, C. R. (2005). Complex interactions among mammalian carnivores in Australia, and their implications for wildlife management. Biological Reviews, 80(3), 387–401. 10.1017/S1464793105006718 16094805

[ece310026-bib-0098] Green, M. , Ganassin, C. , & Reid, D. D. (2009). Report into NSW shark meshing (bather protection) program: incorporating a review of the existing program and environmental assessment. NSW DPI Fisheries Conservation and Aquaculture Branch.

[ece310026-bib-0018] Grueber, C. E. , Nakagawa, S. , Laws, R. J. , & Jamieson, I. G. (2011). Multimodel inference in ecology and evolution: Challenges and solutions. Journal of Evolutionary Biology, 24(4), 699–711. 21272107 10.1111/j.1420-9101.2010.02210.x

[ece310026-bib-0019] Hanf, D. , Hodgson, A. J. , Kobryn, H. , Bejder, L. , & Smith, J. N. (2022). Dolphin distribution and habitat suitability in North Western Australia: Applications and implications of a broad‐scale, non‐targeted dataset. Frontiers in Marine Science, 8, 1–18. 10.3389/fmars.2021.733841 35273967

[ece310026-bib-0020] Hastie, T. J. , & Tibshirani, R. J. (2017). Generalized additive models . 10.1201/9780203753781 8548102

[ece310026-bib-0021] Heithaus, M. (2001a). Predator–prey and competitive interactions between sharks (order Selachii) and dolphins (suborder Odontoceti): A review. Journal of Zoology, 253(1), 53–68. 10.1017/S0952836901000061

[ece310026-bib-0022] Heithaus, M. (2001b). Shark attacks on bottlenose dolphins (*Tursiops aduncus*) in Shark Bay, Western Australia: Attack rate, bite scar frequencies, and attack seasonality. Marine Mammal Science, 17(3), 526–539. 10.1111/j.1748-7692.2001.tb01002.x

[ece310026-bib-0023] Heithaus, M. , Dill, L. , Marshall, G. J. , & Buhleier, B. M. (2002). Habitat use and foraging behavior of tiger sharks (*Galeocerdo cuvier*) in a seagrass ecosystem. Marine Biology, 140, 237–248. 10.1007/S00227-001-0711-7

[ece310026-bib-0024] Heithaus, M. , & Dill, L. M. (2002). Food availability and tiger shark predation risk influence bottlenose dolphin habitat use. Ecology, 83(2), 480–491. 10.1890/0012-9658(2002)083[0480:FAATSP]2.0.CO;2

[ece310026-bib-0025] Heithaus, M. , & Dill, L. M. (2006). Does tiger shark predation risk influence foraging habitat use by bottlenose dolphins at multiple spatial scales? Oikos, 114(2), 257–264. 10.1111/j.2006.0030-1299.14443.x

[ece310026-bib-0026] Heithaus, M. , Kiszka, J. J. , Cadinouche, A. , Dulau‐Drouot, V. , Boucaud, V. , Perez‐Jorge, S. , & Webster, I. (2017). Spatial variation in shark‐inflicted injuries to Indo‐Pacific bottlenose dolphins (*Tursiops aduncus*) of the southwestern Indian Ocean. Marine Mammal Science, 33, 335–341. 10.1111/mms.12346

[ece310026-bib-0027] Heithaus, M. , Wirsing, A. , & Dill, L. (2012). The ecological importance of intact top‐predator populations: A synthesis of 15 years of research in a seagrass ecosystem. Marine and Freshwater Research, 63, 1039–1050. 10.1071/MF12024

[ece310026-bib-0028] Heithaus, M. , Wirsing, A. , Dill, L. , & Heithaus, L. I. (2007). Long‐term movements of tiger sharks satellite‐tagged in Shark Bay, Western Australia. Marine Biology, 151, 1455–1461. 10.1007/S00227-006-0583-Y

[ece310026-bib-0029] Heithaus, M. , Wirsing, A. J. , Burkholder, D. , Thomson, J. , & Dill, L. M. (2009). Towards a predictive framework for predator risk effects: The interaction of landscape features and prey escape tactics. Journal of Animal Ecology, 78(3), 556–562. 10.1111/j.1365-2656.2008.01512.x 19076259

[ece310026-bib-0030] Heupel, M. , & Simpfendorfer, C. (2008). Movement and distribution of young bull sharks *Carcharhinus leucas* in a variable estuarine environment. Aquatic Biology, 1, 277–289. 10.3354/ab00030

[ece310026-bib-0031] Holmes, B. J. , Sumpton, W. D. , Mayer, D. G. , Tibbetts, I. R. , Neil, D. T. , & Bennett, M. B. (2012). Declining trends in annual catch rates of the tiger shark (*Galeocerdo cuvier*) in Queensland, Australia. Fisheries Research, 129–130, 38–45. 10.1016/j.fishres.2012.06.005

[ece310026-bib-0032] Holt, A. R. , Davies, Z. , Tyler, C. , & Staddon, S. (2008). Meta‐analysis of the effects of predation on animal prey abundance: Evidence from UK vertebrates. PLoS One, 3, e2400. 10.1371/journal.pone.0002400 18545690 PMC2405933

[ece310026-bib-0033] Hunt, T. , Bejder, L. , Allen, S. , Rankin, R. , Hanf, D. , & Parra, G. (2017). Demographic characteristics of Australian humpback dolphins reveal important habitat toward the southwestern limit of their range. Endangered Species Research, 32, 71–88. 10.3354/esr00784

[ece310026-bib-0034] Jefferson, T. A. , & Rosenbaum, H. C. (2014). Taxonomic revision of the humpback dolphins (*Sousa* spp.), and description of a new species from Australia. Marine Mammal Science, 30(4), 1494–1541. 10.1111/mms.12152

[ece310026-bib-0035] Jefferson, T. A. , Stacey, P. J. , & Baird, R. W. (1991). A review of killer whale interactions with other marine mammals: Predation to co‐existence. Mammal Review, 21(4), 151–180. 10.1111/j.1365-2907.1991.tb00291.x

[ece310026-bib-0036] Kiszka, J. , Perrin, W. F. , Pusineri, C. , & Ridoux, V. (2011). What drives Island‐associated tropical dolphins to form mixed‐species associations in the Southwest Indian Ocean? Journal of Mammalogy, 92(5), 1105–1111. 10.1644/10-MAMM-A-376.1

[ece310026-bib-0093] Korpimäki, E. (1985). Prey choice strategies of the kestrel Falco tinnunculus in relation to available small mammals and other Finnish birds of prey. Annales Zoologici Fennici, 22(1), 91–104.

[ece310026-bib-0037] Lima, S. L. (1998). Nonlethal effects in the ecology of predator‐prey interactions. Bioscience, 48(1), 25–34. 10.2307/1313225

[ece310026-bib-0038] Lima, S. L. , & Dill, L. M. (1990). Behavioral decisions made under the risk of predation: A review and prospectus. Canadian Journal of Zoology, 68(4), 619–640.

[ece310026-bib-0104] Long, D. , & Jones, R. (1996). White shark predation and scavenging on cetaceans in the Eastern North Pacific Ocean. In P. A. Klimley & D. Ainley (Eds.), Great White Sharks: The Biology of Carcharodon carcharias (pp. 239–307). Academic Press. 10.1016/B978-012415031-7/50028-8

[ece310026-bib-0039] Lowe, C. G. , Wetherbee, B. M. , Crow, G. L. , & Tester, A. L. (1996). Ontogenetic dietary shifts and feeding behavior of the tiger shark, *Galeocerdo cuvier*, in Hawaiian waters. Environmental Biology of Fishes, 47(2), 203–211. 10.1007/BF00005044

[ece310026-bib-0040] Mann, J. , & Barnett, H. (1999). Lethal tiger shark (*Galeocerdo cuvier*) attack on bottlenose dolphin (*Tursiops* sp.) calf: Defense and reactions by the mother. Marine Mammal Science, 15(2), 568–575.

[ece310026-bib-0041] Martin, R. A. , & Hammerschlag, N. (2012). Marine predator–prey contests: Ambush and speed versus vigilance and agility. Marine Biology Research, 8(1), 90–94. 10.1080/17451000.2011.614255

[ece310026-bib-0042] Melillo‐Sweeting, K. M. , Maust‐Mohl, M. , & Smukall, M. J. (2021). Examining shark bite scars on dolphins off Bimini, The Bahamas: Comparisons between bottlenose and Atlantic spotted dolphins. Marine Mammal Science, 1(11), 18–28. 10.1111/mms.12840

[ece310026-bib-0097] Melillo‐Sweeting, K. M. , Turnbull, S. D. , & Guttridge, T. L. (2014). Evidence of shark attacks on Atlantic spotted dolphins (*Stenella frontalis*) off Bimini. The Bahamas. Marine Mammal Science, 30(3), 1158–1164. 10.1111/mms.12082

[ece310026-bib-0043] Menge, B. A. (1976). Organization of the New England rocky intertidal community: Role of predation, competition, and environmental heterogeneity. Ecological Monographs, 46(4), 355–393. 10.2307/1942563

[ece310026-bib-0044] Meyer, C. , Clark, T. , Papastamatiou, Y. , Whitney, N. , & Holland, K. (2009). Long‐term movement patterns of tiger sharks *Galeocerdo cuvier* in Hawaii. Marine Ecology Progress Series, 381, 223–235. 10.3354/MEPS07951

[ece310026-bib-0045] Microsoft Corporation . (2022). Microsoft excel . https://office.microsoft.com/excel

[ece310026-bib-0046] Mills, M. G. L. , & Shenk, T. M. (1992). Predator‐Prey Relationships: The Impact of Lion Predation on Wildebeest and Zebra Populations. Journal of Animal Ecology, 61(3), 693–702. 10.2307/5624

[ece310026-bib-0099] Monteiro, M. S. , Vaske, T. Jr , Barbosa, T. M. , & de Alves, M. D. O. (2006). Predation by a shortfin mako shark, *Isurus oxyrinchus*, Rafinesque, 1810, on a pantropical spotted dolphin, *Stenella attenuata*, calf in Central Atlantic waters. Latin American Journal of Aquatic Mammals, 5(2), 141–144. 10.5597/lajam00106

[ece310026-bib-0047] Myers, R. A. , Baum, J. L. , Shepherd, T. D. , Powers, S. P. , & Peterson, C. H. (2007). Cascading effects of the loss of apex predatory sharks from a coastal ocean. Science, 315(5820), 1846–1850. 10.1126/science.1138657 17395829

[ece310026-bib-0048] Naimi, B. (2015). USDM: Uncertainty analysis for species distribution models. R package version 1.1‐15. *R Documentation*. http://www.rdocu‐mentation.org/packages/usdm

[ece310026-bib-0094] Norrdahl, K. , & Korpimäki, E. (1995). Effects of predator removal on vertebrate prey populations: birds of prey and small mammals. Oecologia, 103(2), 241–248. 10.1007/BF00329086 28306779

[ece310026-bib-0049] Norris, K. S. , & Dohl, T. P. (1980). Behavior of the Hawaiian spinner dolphin. Fishery Bulletin, 77(4), 821.

[ece310026-bib-0050] Parra, G. , & Cagnazzi, D. (2015). Conservation status of the Australian humpback dolphin (*Sousa sahulensis*) using the IUCN Red List Criteria. Advances in Marine Biology, 73, 157–192. 10.1016/bs.amb.2015.07.006 26790892

[ece310026-bib-0051] Parra, G. J. , Cagnazzi, D. , & Beasley, I. (2017). *Orcaella heinsohni* (errata version published in 2018). The IUCN Red List Of Threatened Species 2017: e. T136315A123793740. *IUCN Red List of Threatened Species*. 10.2305/IUCN.UK.2017-3.RLTS.T136315A50385982.en

[ece310026-bib-0052] Parra, G. J. , Cagnazzi, D. , Perrin, W. , & Braulik, G. T. (2017). *Sousa sahulensis*. The IUCN Red List of Threatened Species 2017: e.T82031667A82031671. *IUCN Red List of Threatened Species*. 10.2305/IUCN.UK.2017-3.RLTS.T82031667A82031671.en

[ece310026-bib-0053] Parra, G. J. , Corkeron, P. J. , & Arnold, P. (2011). Grouping and fission–fusion dynamics in Australian snubfin and Indo‐Pacific humpback dolphins. Animal Behaviour, 82(6), 1423–1433. 10.1016/j.anbehav.2011.09.027

[ece310026-bib-0054] Parra, G. J. (2006). Resource partitioning in sympatric delphinids: Space use and habitat preferences of Australian snubfin and Indo‐Pacific humpback dolphins. Journal of Animal Ecology, 75(4), 862–874. 10.1111/j.1365-2656.2006.01104.x 17009750

[ece310026-bib-0055] Parra, G. J. , Cagnazzi, D. , Jedensjö, M. , Ackermann, C. , Frere, C. , Seddon, J. , Nikolic, N. , & Krützen, M. (2018). Low genetic diversity, limited gene flow and widespread genetic bottleneck effects in a threatened dolphin species, the Australian humpback dolphin. Biological Conservation, 220, 192–200. 10.1016/j.biocon.2017.12.028

[ece310026-bib-0056] Parra, G. J. , Corkeron, P. J. , & Marsh, H. (2006). Population sizes, site fidelity and residence patterns of Australian snubfin and Indo‐Pacific humpback dolphins: Implications for conservation. Biological Conservation, 129(2), 167–180. 10.1016/j.biocon.2005.10.031

[ece310026-bib-0103] Paterson, R. A. (1990). Effects of long‐term anti‐shark measures on target and non‐target species in Queensland, Australia. Biological Conservation, 52(2), 147–159. 10.1016/0006-3207(90)90123-7

[ece310026-bib-0057] Preisser, E. L. , Bolnick, D. I. , & Benard, M. F. (2005). Scared to death? The effects of intimidation and consumption in predator–prey interactions. Ecology, 86(2), 501–509.

[ece310026-bib-0058] R Core Team . (2022). R: The R project for statistical computing . https://www.r‐project.org/

[ece310026-bib-0059] Ritchie, E. G. , & Johnson, C. N. (2009). Predator interactions, mesopredator release and biodiversity conservation. Ecology Letters, 12(9), 982–998. 10.1111/j.1461-0248.2009.01347.x 19614756

[ece310026-bib-0060] Robertson, A. I. , & Duke, N. C. (1987). Mangroves as nursery sites: Comparisons of the abundance and species composition of fish and crustaceans in mangroves and other nearshore habitats in tropical Australia. Marine Biology, 96(2), 193–205. 10.1007/BF00427019

[ece310026-bib-0061] Robertson, A. I. , & Duke, N. C. (1990). Mangrove fish‐communities in tropical Queensland, Australia: Spatial and temporal patterns in densities, biomass and community structure. Marine Biology, 104(3), 369–379. 10.1007/BF01314339

[ece310026-bib-0062] Rosenzweig, M. L. (1981). A theory of habitat selection. Ecology, 62(2), 327–335.

[ece310026-bib-0063] Schmitz, O. J. , Hambäck, P. A. , & Beckerman, A. P. (2000). Trophic cascades in terrestrial systems: A review of the effects of carnivore removals on plants. The American Naturalist, 155(2), 141–153. 10.1086/303311 10686157

[ece310026-bib-0064] Scott, E. M. , Mann, J. , Watson‐Capps, J. J. , Sargeant, B. L. , & Connor, R. C. (2005). Aggression in bottlenose dolphins: Evidence for sexual coercion, male‐male competition, and female tolerance through analysis of tooth‐rake marks and behaviour. Behaviour, 142(1), 21–44.

[ece310026-bib-0065] Simpfendorfer, C. A. (1992). Biology of Tiger sharks (*Galeocerdo cuvier*) caught by the Queensland shark meshing program off Townsville, Australia. Marine and Freshwater Research, 43(1), 33. 10.1071/MF9920033

[ece310026-bib-0066] Simpfendorfer, C. A. , & Milward, N. E. (1993). Utilisation of a tropical bay as a nursery area by sharks of the families Carcharhinidae and Sphyrnidae. Environmental Biology of Fishes, 37(4), 337–345. 10.1007/BF00005200

[ece310026-bib-0067] Smith, F. , Allen, S. J. , Bejder, L. , & Brown, A. M. (2018). Shark bite injuries on three inshore dolphin species in tropical northwestern Australia. Marine Mammal Science, 34(1), 87–99. 10.1111/mms.12435

[ece310026-bib-0068] Spiess, A.‐N. (2018). Package ‘qpcR’. *Modelling and analysis of real‐time PCR data*. https://CRAN.R‐project.org/web/packages/qPCR/qPCR.pdf

[ece310026-bib-0069] Stacey, P. J. , & Leatherwood, S. (1997). The Irrawaddy dolphin, *Orcaella brevirostris*: A summary of current knowledge and recommendations for conservation action. Asian Marine Biology, 14, 195–214.

[ece310026-bib-0070] Stein, R. A. (1977). Selective predation, optimal foraging, and the predator‐prey interaction between fish and crayfish. Ecology, 58(6), 1237–1253. 10.2307/1935078

[ece310026-bib-0071] Turesson, H. , & Brönmark, C. (2007). Predator–prey encounter rates in freshwater piscivores: Effects of prey density and water transparency. Oecologia, 153(2), 281–290. 10.1007/s00442-007-0728-9 17453254

[ece310026-bib-0072] Turnbull, S. D. , & Dion, D. (2012). White shark (*Carcharodon carcharias*) attack on a harbor porpoise (*Phocaena phocaena*) in the Bay of Fundy, Canada. Northeastern Naturalist, 19(4), 705–707.

[ece310026-bib-0073] Vance, R. R. (1979). Effects of grazing by the sea urchin, *Centrostephanus coronatus*, on prey community composition. Ecology, 60(3), 537–546. 10.2307/1936074

[ece310026-bib-0074] Webb, P. (2021). Introduction to oceanography. Roger Williams University.

[ece310026-bib-0095] Wilkinson, K. A. , Wells, R. S. , Pine, W. E. , & Borkhataria, R. R. (2017). Shark bite scar frequency in resident common bottlenose dolphins (*Tursiops truncatus*) in Sarasota Bay. Florida. Marine Mammal Science, 33(2), 678–686. 10.1111/mms.12385

[ece310026-bib-0075] Wirsing, A. , Heithaus, M. , & Frid, A. (2014). Cross‐fertilizing aquatic and terrestrial research to understand predator risk effects. Wiley Interdisciplinary Reviews: Water, 1, 439–448. 10.1002/WAT2.1039

[ece310026-bib-0076] Wirsing, A. , & Ripple, W. (2011). A comparison of shark and wolf research reveals similar behavioral responses by prey. Frontiers in Ecology and the Environment, 9, 335–341. 10.1890/090226

[ece310026-bib-0077] Wood, S. N. (2001). Mgcv: GAMs and generalized ridge regression for R. R News, 1(2), 20–25.

[ece310026-bib-0102] Wood, S. N. (2017). Generalized Additive Models: An Introduction with R (2nd ed.). Chapman and Hall/CRC. 10.1201/9781315370279

[ece310026-bib-0100] Würsig, B. , & Jefferson, T. A. (1990). Methods of photo‐identification for small cetaceans. Reports of the International Whaling Commission. Special, (12), 43–52.

[ece310026-bib-0078] Würsig, B. , & Würsig, M. (1977). The photographic determination of group size, composition, and stability of coastal porpoises (*Tursiops truncatus*). Science, 198(4318), 755–756. 10.1126/science.198.4318.755

